# Low detection rate of RT-PCR-confirmed COVID-19 using IgM/IgG rapid antibody tests in a large community sample in Lima, Peru

**DOI:** 10.1186/s12879-023-08003-7

**Published:** 2023-02-02

**Authors:** Stephanie Law, Marco A. Tovar, Molly F. Franke, Roger Calderon, Santiago Palomino, Gissella Valderrama, Fernando Llanos, Gustavo E. Velásquez, Carole D. Mitnick, Leonid Lecca

**Affiliations:** 1 Department of Global Health and Social Medicine, Harvard Medical School, Boston, MA USA; 2 Department of Medicine, McGill University, Montreal, QC Canada; 3 Socios En Salud Sucursal Peru, Lima, Perú; 4 Escuela de Medicina, Facultad de Ciencias de la Salud, Universidad Peruana de Ciencias Aplicadas, Lima, Peru; 5 Dirección de Redes Integradas de Salud Lima Norte (DIRIS Lima Norte), Lima, Perú; 6 Departamento Académico de Salud Pública, Administración y Ciencias Sociales, Universidad Peruana Cayetano Heredia, Lima, Perú; 7 UCSF Center for Tuberculosis, University of California, San Francisco, San Francisco, CA USA; 8 Division of HIV, Infectious Diseases, and Global Medicine, University of California, San Francisco, San Francisco, CA USA; 9 Division of Global Health Equity, Brigham and Women’s Hospital, Boston, MA USA

**Keywords:** COVID-19, SARS-CoV-2 antibody testing, Peru

## Abstract

**Background:**

Rapid IgM/IgG antibody tests were largely used in lieu of RT-PCR tests as part of COVID-19 public health response activities in Lima, Peru. To assess their utility, we explored the relationship between the time since onset of several COVID-19-related symptoms and the sensitivity of a rapid combined IgM/IgG antibody test.

**Methods:**

We collected data from a community sample of individuals (n = 492) who received concurrent RT-PCR and rapid IgM/IgG antibody testing between May 2020 and March 2021. We estimated the sensitivity of the antibody test, against the RT-PCR test, by weeks since symptom onset via segmented regression analysis.

**Results:**

The overall sensitivity of the rapid IgM/IgG antibody test was 46.7% (95% CI, 42.4–51.2%). Among 372 (75.6%) participants who reported COVID-19-related symptoms, sensitivity increased from 30.4% (95% CI, 24.7–36.6%) in week 1 after symptom onset to 83.3% (95% CI, 41.6–98.4%) in week 4. The test sensitivity increased by 31.9% (95% CI, 24.8–39.0%) per week until week 2 to 3, then decreased by − 6.0% (95% CI, − 25.7–13.7%) per week thereafter.

**Conclusion:**

Rapid antibody tests are a poor substitute for RT-PCR testing, regardless of presenting symptoms. This highlights the need for future pandemic planning to include timely and equitable access to gold-standard diagnostics, treatment, and vaccination.

**Supplementary Information:**

The online version contains supplementary material available at 10.1186/s12879-023-08003-7.

## Background

The most reliable diagnostic test for SARS-CoV-2—the virus that causes COVID-19—is the real-time reverse transcription polymerase chain reaction (RT-PCR) test. The test is often performed on nasopharyngeal swabs and increasingly, on anterior nasal swabs and deep saliva samples. The rapid, serological-based antibody test has also been adopted in many settings. Instead of directly detecting the virus itself, which the RT-PCR test does, the point-of-care antibody test assesses the host’s immune response to the virus by detecting the presence (or absence) of antibodies against SARS-CoV-2.

Growing evidence suggests that the serological antibody test and the RT-PCR test have different detection time windows. Generally, RT-PCR can detect viral RNA starting at symptom onset and peaks after one week; its sensitivity slowly wanes over the ensuing five [1]. On the other hand, antibody test sensitivity tends to be low in the first week after symptom onset. It increases during the second week, peaks in the third week and remains high at least through the first 3 months [2, 3]. Thus, serological antibody tests might not be suitable as a primary diagnostic tool, but rather one that complements the RT-PCR test. However, most available evidence on the sensitivity of rapid antibody tests has relied on small laboratory samples and hospitalized COVID-19 patients. It may not, therefore, be applicable to community settings and a broader range of clinical presentations including persons with COVID-19 who present with milder symptoms, or none at all [2]. Additionally, the specific antibody isotype - IgA, IgM, IgG, or a combination - assayed affect test sensitivity, and there is wide variability in the sensitivity of commercially available test assays [1, 2].

Despite its shortcomings, the serological antibody test was used as the primary diagnostic tool during the first wave of COVID-19 in Peru, and it continues to be used today even as other tests are becoming more available. Peru is one of the countries hardest-hit by the COVID-19 pandemic, where there have been over 2.0 million cases and nearly 200,000 deaths by late August 2021 [4]. In order to inform the use of the antibody test and to examine its sensitivity in a greater clinical setting, we sought to explore the relationship between the time since onset of several important COVID-19 symptoms (e.g., fever, cough, sore throat, difficulty breathing) and the sensitivity of rapid serologic antibody tests among a Peruvian sample of individuals with a diagnosis of COVID-19 based on a concurrent positive SARS-CoV-2 RT-PCR test.

## Method

### Study setting

Peru has been heavily affected by the SARS-CoV-2 pandemic, particularly in its capital city, Lima. From the start of its COVID-19 public health response, through December 2020 at least, SARS-CoV-2 rapid antibody testing was the predominant diagnostic test used in public clinics, with rapid antigen and RT-PCR testing less frequently available. We enrolled participants from the northern region of Lima (Dirección de Redes Integradas de Salud Lima Norte, also known as DIRIS Lima Norte), which includes urban and peri-urban areas, informal human settlements, and high rates of poverty.

### Data collection

We used data collected as part of COVID-19 public health response activities carried out in Lima, Peru, between May 2020 and March 2021, by Socios En Salud (SES) and the Peruvian Ministry of Health (MoH) in DIRIS Lima Norte.

Between May and July 2020, all close contacts of newly diagnosed COVID-19 patients received SARS-CoV-2 antibody testing via home visits by SES teams. As primary care facilities began reopening in August 2020, SES teams started conducting facility-based SARS-CoV-2 antibody testing in individuals attending the clinic who reported any comorbidities or risk factors for COVID-19.

Prior to testing, all individuals completed a short survey that, among other variables, collected sociodemographic information and a detailed history of COVID-19-related symptoms (including start and end dates, when applicable). The specific symptoms probed in the questionnaire were: cough, sore throat, fever, nasal congestion, difficulty breathing, malaise, diarrhea, vomiting and headache. Respondents were also asked if they had any other symptoms not included in that list. Among the list of other symptoms reported, loss of smell or taste were specifically extracted and included in our analysis.

All SARS-CoV-2 antibody testing was performed using the Standard Q COVID-19 IgG/IgM Duo (SD Biosensor, Suwon, Korea). If the result of the rapid SARS-CoV-2 antibody screening test was indeterminate, a second test was performed. A positive rapid antibody test was defined as any of the following: reactive for IgG, IgM, or both IgM and IgG. Those with a reactive result for IgG and/or IgM were managed according to MoH guidelines, which included isolation, virtual clinical follow-up, and contact tracing.

Due to limited availability, SARS-CoV-2 RT-PCR tests were reserved for specific subgroups during the study period. From May to August 2020, symptomatic household contacts of individuals diagnosed with COVID-19 received RT-PCR testing. Then from August to October health-care workers involved in the COVID response were RT-PCR tested routinely every two weeks, irrespective of symptoms. From August to mid-November, individuals who had any chronic diseases or who were pregnant - with or without any COVID-19 symptoms - received RT-PCR testing. From mid-November onwards, asymptomatic household contacts of individuals diagnosed with COVID-19 and anyone with 3 or more symptoms - in addition to those qualifying under the preceding eligibility criteria - received RT-PCR testing.

For RT-PCR testing, nasopharyngeal swabs were placed in viral transport media (VTM) and maintained in cold chain to laboratory transfer for RNA extraction. The genesig® Real-Time PCR Coronavirus COVID-19 (Primerdesign Ltd, UK), targeted to the RNA polymerase (RdRp) gene of SARS-CoV-2, was used for SARS-CoV-2 RNA detection according to the manufacturer’s instructions.

In the current analysis, we included persons enrolled by the SES COVID-19 team who had their first positive SARS-CoV-2 RT-PCR test result from a sample collected between June 7, 2020, and March 18, 2021, and whose blood sample for a rapid antibody test was collected on the same day as the sample collected for the SARS-CoV-2 RT-PCR test. We excluded anyone who had a prior positive SARS-CoV-2 RT-PCR, antibody, or antigen test. If a person had more than one RT-PCR positive test performed with a matched antibody test, only the first one was included.

### Data analysis

All statistical analyses were performed using R 4.0.0 (R Foundation for Statistical Computing, Vienna, Austria) with a 5% significance level for hypothesis testing. The time since onset of each symptom was defined as the time from the reported start date to the time of sample collection for the paired RT-PCR and rapid antibody tests. The symptom start date was defined as the most recent start date reported by an individual for a given symptom prior to the date of RT-PCR and antibody tests. Start dates for symptoms that had resolved before the test date were included in the analysis. In aggregate symptom analysis, the date of symptom onset was determined by the earliest reported symptom. The primary analysis excluded extreme outliers; i.e., persons whose time since symptom onset was longer than the third quartile plus 3 times the interquartile range (IQR, defined as the difference between the first and third quartile of the reported times since onset for a given symptom). In sensitivity analyses, we explored whether results were sensitive to the exclusion of mild outliers only (i.e., beyond the third quartile plus 1.5 times the IQR) or inclusion of all outliers (Additional file 1: Tables S1, S2).

Among RT-PCR-positive persons, we compared demographic and symptom data between those with and without a reactive rapid antibody test. We estimated the sensitivity of the antibody test across different subgroups according to time since onset of symptoms prior to testing. Sensitivity of the antibody test was defined as the probability of a reactive rapid antibody test among those with a positive RT-PCR test. We estimated 95% confidence intervals (CI) for proportions via the Wilson score method. For comparisons, we estimated *p* values using the chi-squared test for proportions and the Welch t-test for means. We also stratified the analyses according to the targeted antibody isotype (i.e., IgG only, IgM only, or reactive on either or both).

We performed segmented linear regression analysis [5], weighted by sample size, to estimate the change in test sensitivity per week since symptom onset. The segmented linear regression model estimated a breakpoint (i.e., a change in slope) if it improved the model fit; otherwise, no breakpoint was estimated. Additionally, we repeated the analyses for ten common COVID-19 symptoms, individually: cough, sore throat, fever, nasal congestion, difficulty breathing, malaise, diarrhea, vomiting, headache and loss of taste or smell.

## Findings

### Overview

**Fig. 1:**
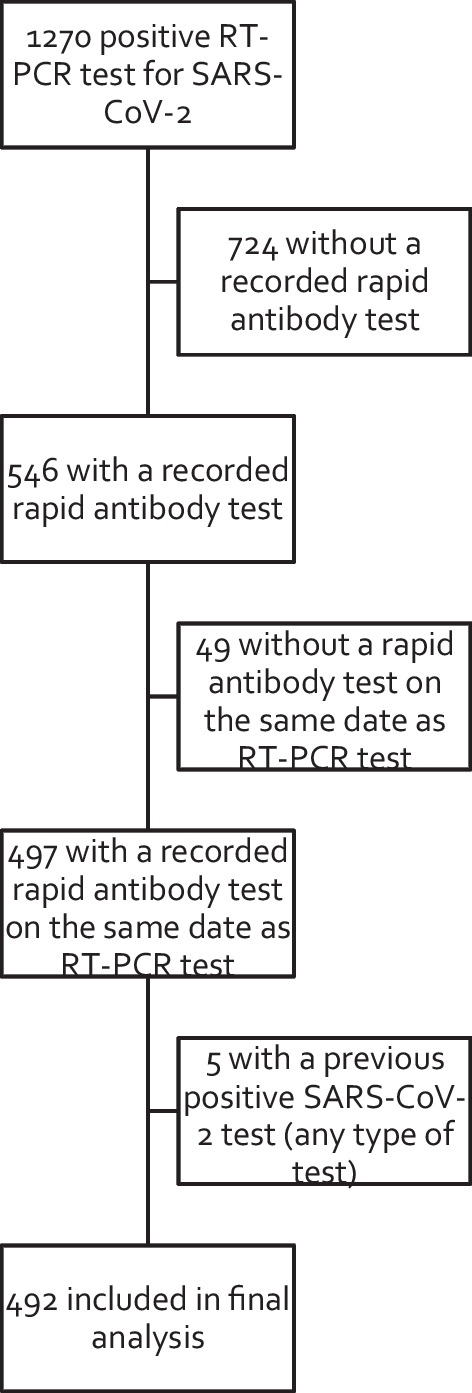
Study flowchart of individuals included in final analysis

During the inclusion period (June 7, 2020 to March 18, 2021), 1270 persons tested positive for SARS-CoV-2. Of these, 492 individuals (38.6%) met the inclusion criteria and were included in this analysis (see Fig. 1 for a study flowchart). Mean age was 48.4 ± 20.1 years. About half were female (n = 257, 52.2%) and half reported having had contact with someone diagnosed with COVID-19 in the previous two weeks (n = 249, 50.6%). Two hundred thirty (46.7%) individuals had a reactive rapid antibody test result to either or both IgM and IgG; of these, 51 (22.2%) tested positive for IgM only, 50 (21.7%) for IgG only, 129 (56.1%) for both IgM and IgG.

### Frequency and time since onset of symptoms

The primary analysis included 372 (75.6%) persons who reported at least one symptom prior to sample collection for the diagnostic tests (Table 1), after excluding extreme outliers. The proportions reporting symptoms were similar between those with a reactive or negative rapid antibody test result (p = 0.11). Those with a negative rapid antibody test reported having a fever more frequently than those with a reactive result (p = 0.01); there were no other statistically significant differences in symptom frequency between the two groups. Results were similar in sensitivity analysis that excluded no participants (Additional file 1: Table S1); in the sensitivity analysis that excluded extreme and mild outliers, a larger proportion of those with a negative antibody test reported having any symptoms (n = 205, 78.2%) compared to those with a reactive test (n = 153, 66.5%), as well as having a sore throat, fever, congestion and malaise (p < 0.05, Additional file 1: Table S2). After exclusion of both extreme and mild outliers, there were no participants who had had more than 3 weeks of symptoms prior to sample collection.

**Table 1:** Characteristics of participants with sample collected (and tested) for serologic antibody test on same day as sample that resulted in RT-PCR positive for SARS-CoV-2 (n = 492)

Characteristic	Total (n = 492)	Negative antibody test (n = 262)	Positive antibody test (n = 230)	p–value^a^
Age, mean (SD)	48.4 (20.1)	47.0 (21.1)	49.9 (18.9)	0.10
Female, n (%)^b^	257 (52.2)	126 (48.1)	131 (57.0)	0.07
Any contact history, n (%)	249 (50.6)	127 (48.5)	122 (53.0)	0.36
Reported having any symptoms, n (%)	372 (75.6)	206 (78.6)	166 (72.2)	0.12
Cough	244 (49.6)	132 (50.4)	112 (48.7)	0.78
Sore throat	180 (36.6)	105 (40.1)	75 (32.6)	0.10
Fever	171 (34.8)	106 (40.5)	65 (28.3)	0.01
Nasal congestion	127 (25.8)	77 (29.4)	50 (21.7)	0.07
Difficulty breathing	61 (12.4)	26 (9.9)	35 (15.2)	0.10
Malaise	179 (36.4)	103 (39.3)	76 (33.0)	0.18
Diarrhea	65 (13.2)	34 (13.0)	31 (13.5)	0.98
Vomiting	52 (10.6)	23 (8.8)	29 (12.6)	0.22
Headache	162 (32.9)	90 (34.4)	72 (31.3)	0.53
Loss of taste or smell	27 (5.5)	12 (4.6)	15 (6.5)	0.46
Median number of days since onset of symptoms, among those reporting symptoms (range) (N = 372)	7 (1–31)	5 (1–31)	9 (2–31)	< 0.01
Cough (n = 244)	7 (1–24)	5 (1–24)	8 (2–24)	< 0.01
Sore throat (n = 180)	6 (1–28)	5 (1–18)	9 (2–28)	< 0.01
Fever (n = 171)	4 (1–22)	3.5 (1–18)	7 (1–22)	< 0.01
Nasal congestion (n = 127)	6 (1–31)	4.0 (1–31)	9 (1–31)	< 0.01
Difficulty breathing (n = 61)	7 (1–29)	4 (1–15)	8 (2–29)	< 0.01
Malaise (n = 179)	6 (1–24)	5 (1–19)	8 (1–24)	< 0.01
Diarrhea (n = 65)	6 (1–24)	3 (1–24)	7 (1–24)	< 0.01
Vomiting (n = 52)	4 (0–24)	3 (1–9)	7 (2–21)	< 0.01
Headache (n = 162)	5 (1–21)	5 (1–19)	7 (1–21)	< 0.01
Loss of taste or smell (n = 27)	5 (2–16)	3.5 (3–10)	7 (2–16)	0.03

^a^Wilcoxon-Mann-Whitney Test for comparing medians, Pearson’s chi-squared test for comparing proportions. ^b^Two people with a negative rapid antibody test result were missing sex data

Among those who reported symptoms, the median time since onset of the first reported symptom was 7 (range: 1 to 31) days. It was significantly longer (p < 0.01) among those with a reactive rapid antibody test (median = 9, range: 2 to 31) days than among those with a negative test (median = 5, range: 1 to 31) days (Table 1). In terms of individual symptoms, the median time since onset of every symptom was significantly longer for the group with a reactive antibody test.

### Sensitivity of the rapid antibody test

**Fig. 2:**
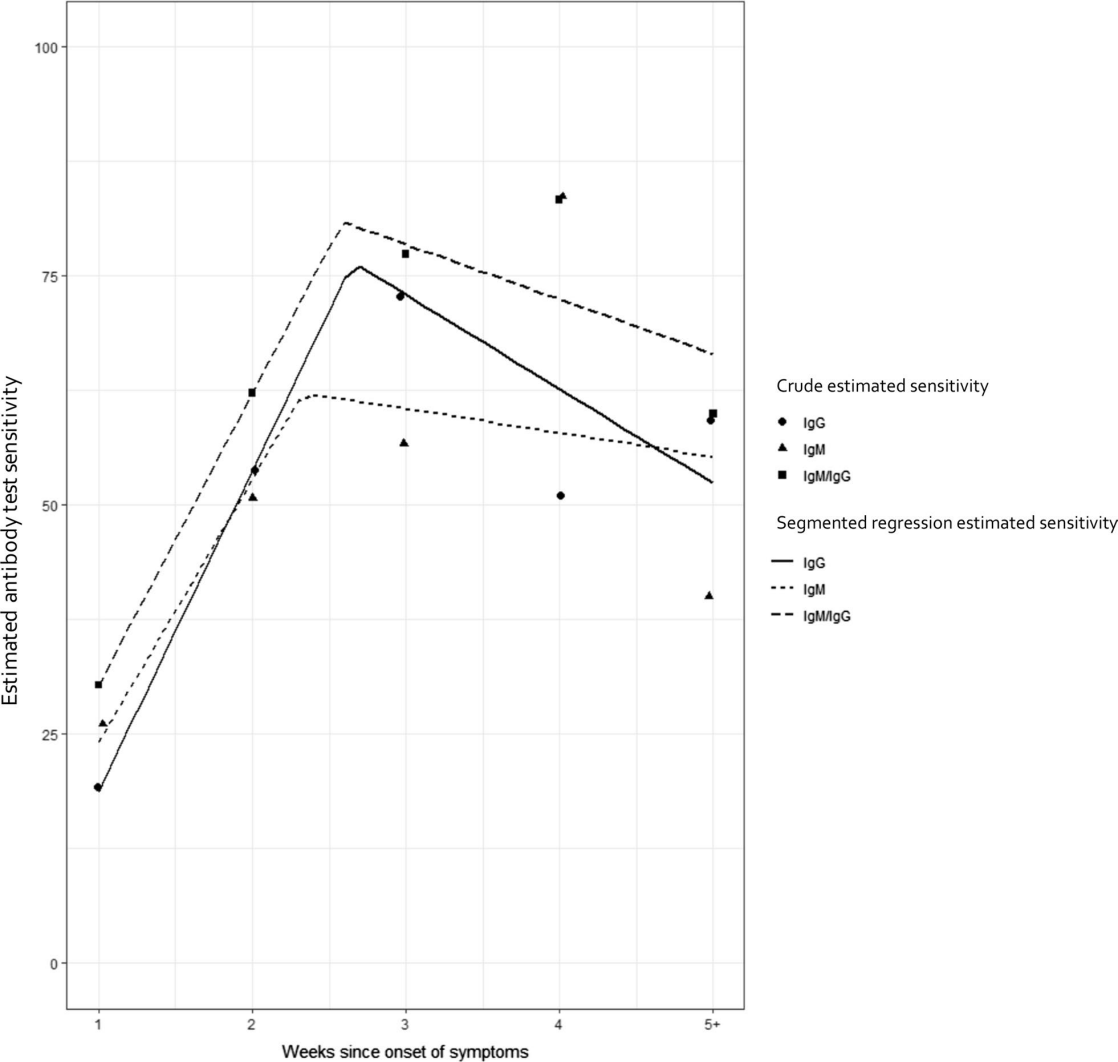
Estimated sensitivity (crude and from segmented regression) of the SARS-CoV-2 rapid antibody versus weeks since onset of symptoms among those who reported at least one symptom before testing, stratified by targeted antibody isotype*, using the RT-PCR test as the reference

^*^IgM/IgG antibody test positivity included those who had either or both IgM and IgG band; IgM antibody test positivity included those who had at least an IgM band; IgG antibody test positivity included those who had at least IgG band.

Using RT-PCR as the reference standard, the overall sensitivity of the rapid antibody test was 46.7% (95% CI, 42.4–51.2%) (Table 2). There was no difference in the sensitivity across antibody isotypes. Among those who reported symptoms, the sensitivity of the antibody test increased from 30.4% (95% CI, 24.7–36.6%) in week 1 since symptom onset, to 83.3% (95% CI, 41.6–98.4%) in week 4 (Table 2; Fig. 2). The segmented regression analysis estimated that the test sensitivity increased by 31.9% (95% CI, 24.8–39.0%) per week until its peak at week 2.6 (95% CI, 2.1 to 3.0 weeks), then decreased by − 6.0% (95% CI, − 25.7–13.7%) per week thereafter.

**Table 2:** Sensitivity of the rapid antibody test, stratified by antibody isotype

Group	Sensitivity of the IgM/IgG antibody isotype, % (95% CI)	Sensitivity of the IgM antibody isotype, % (95% CI)	Sensitivity of the IgG antibody isotype, % (95% CI)	IgM vs. IgG isotype, p–value
Full sample (n = 492)	46.7 (42.4, 51.2)	36.6 (32.5, 40.9)	36.4 (32.3, 40.7)	1.0
Presence of reported symptoms				
Yes (n = 372)	44.6 (39.7, 49.7)	36.3 (31.6, 41.3)	34.4 (29.8, 39.4)	0.65
No (n = 120)	53.3 (44.4, 62.0)	37.5 (29.4, 46.4)	42.5 (34.0, 51.4)	0.51
Yes vs. no, p–value	0.12	0.90	0.14	n/a
Time since onset of earliest symptom, among those reporting symptoms (N = 372)				
Week 1 (n = 224)	30.4 (24.7, 36.6)	24.1 (19.0, 30.1)	18.8 (14.2–24.4)	0.21
Week 2 (n = 106)	62.3 (52.7, 70.9)	52.8 (43.4, 62.1)	53.8 (44.3–63.0)	1.0
Week 3 (n = 31)	77.4 (59.8, 88.8)	58.1 (40.7, 73.5)	74.2 (56.5–86.4)	0.28
Week 4 (n = 6)	83.3 (41.6, 98.4)	83.3 (41.6, 98.4)	50.0 (19.0–81.0)	0.54
Week 5+ (n = 5)	60.0 (23.1, 88.0)	40.0 (12.0–76.9)	60.0 (23.1–88.0)	1.0
Comparing time intervals, p–value	< 0.01	< 0.01	< 0.01	n/a

IgM/IgG antibody test positivity included those who had either or both IgM and IgG band; IgM antibody test positivity included those who had at least an IgM band; IgG antibody test positivity included those who had at least an IgG band. The p-values were estimated using Pearson’s chi-squared test

Results were similar on aggregate and dynamic sensitivity over time (Additional file 1: Fig. S1 and Table S1) in the sensitivity analysis in which no outliers were excluded. However, after removal of extreme and mild outliers, the difference in sensitivity of the antibody test between people with symptoms (42.7% [95% CI, 37.7%, 47.9%]) and without symptoms (57.5% [95% CI, 49.0%, 65.5%]) was statistically significant (p < 0.01; Additional file 1: Table S3).

**Fig. 3:**
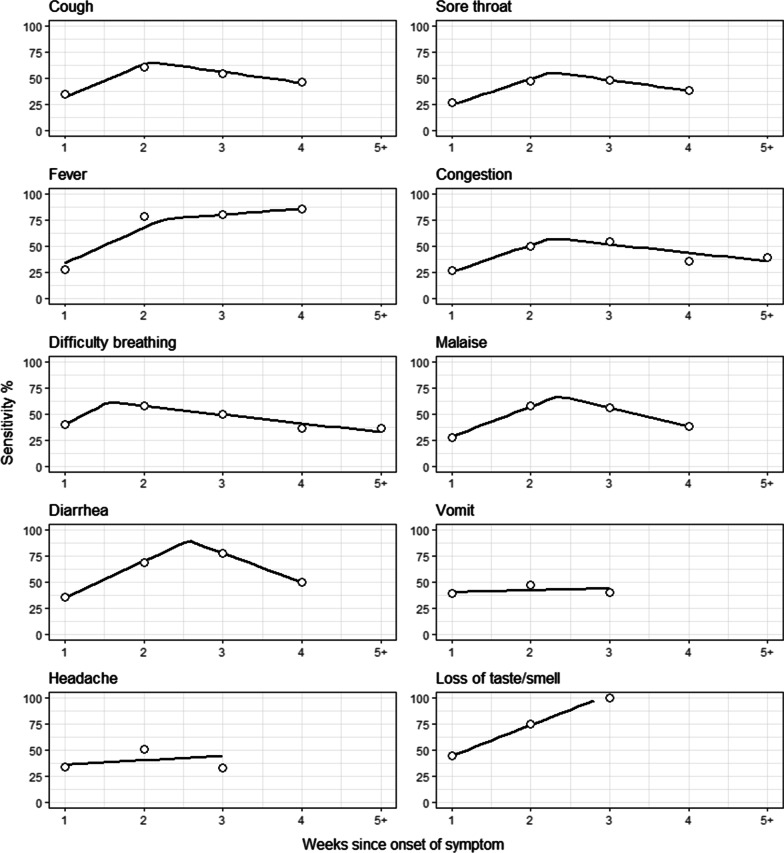
Sensitivity of the SARS-CoV-2 rapid antibody versus weeks since onset of individual symptoms among those who reported the symptom, using the RT-PCR test as the reference. The circles show the crude estimated sensitivity. The lines show the fitted segmented regression

Antibody test sensitivity, and its change over time, varied widely by individual symptoms (Fig. 3, Additional file 1: Table S5). In the first week since onset of each symptom, the test sensitivity ranged from 26.4% (95% CI, 19.5–34.6%) for having a sore throat to 44.4% (95% CI, 24.6–66.3%) for experiencing a loss of taste or smell. The antibody test sensitivity peaked in weeks 2 or 3 after onset of nearly all symptoms - except having a fever; for those who reported a fever, the test sensitivity peaked at week 4 at approximately 85.7% (95% CI, 46.4–99.0%). In the segmented regression analyses, the initial change in antibody test sensitivity ranged from 15.2% (95% CI, 10.7–19.7%) to 39.9% (95% CI 35.8–44.0%) per week (all p < 0.05; Additional file 1: Table S6). The median estimated breakpoint was at week 2.3 (range from week 1.5 (95% CI, 1.4 to 1.7) to week 2.6 (95% CI, 2.4 to 2.7) after symptom onset; there was no estimated breakpoint for vomiting, headache or loss of taste or smell.

## Discussion

Our study examined the utility of the Standard Q COVID-19 IgG/IgM Duo test against SARS-CoV-2, in a non-hospitalized community sample of individuals with RT-PCR-confirmed COVID-19 in Lima, Peru. In 492 individuals, the overall sensitivity of the rapid antibody test was 46.7% (95% CI, 42.4–51.2%). The sensitivity of the rapid IgM/IgG antibody test increased over time, particularly during the first two to three weeks after the onset of symptoms, which is in agreement with a published systematic review by Bastos et al. [3]. Importantly, our analysis suggests the antibody test sensitivity likely increases in the first two or three weeks at a rate of approximately 31.9% (95% CI, 24.8%to 39.0%), peaks at 2.6 weeks, and then gradually declines. This finding was robust across our sensitivity analyses, which either excluded additional mild outliers or included all outliers.

We found some variability in the increase in antibody test sensitivity over time since onset of individual symptoms. For example, the sensitivity of the antibody test among those with a fever rose more precipitously from the first week, at 27.9% (95% CI, 21.1–36.1%), to the second week, at 78.4% (95% CI, 62.5–88.8%) after onset, than did most other symptoms. In nearly all cases, the test sensitivity either plateaued or decreased after two weeks. The test sensitivity was below 40% for all symptoms during the first week of onset except loss of taste and smell (44.4% [95% CI, 24.6–66.3%]). Possible explanations include that this symptom may arise later in the usual course of disease, which may allow more time for the body to produce more antibodies [6], and there may be better recall for self-reported loss of taste and smell, which is more commonly - and specifically - associated with COVID-19 disease. More research would be needed to confirm this finding, as there were only 27 (5.5%) individuals who reported a loss of taste or smell.

Although differences in peaks of isotype sensitivity were not statistically significantly different, our data suggested enhanced sensitivity of IgG in week 3 (74.2% [95% CI, 56.5–86.4%]) and of IgM sensitivity in week 4 (83.3% [95% CI 41.6–98.4%]). This confirms earlier studies reporting higher sensitivity with tests targeting the IgM antibody isotypes [7]. Moreover, evaluation of both isotypes resulted in sensitivity above 60% even in week 2; this was not achievable considering only IgG or IgM. If the objective is to identify individuals who require (support for isolation) or care, use of an antibody test that targets both isotypes is critical. We did not include a test with IgA so cannot comment on the potential additional contribution of testing for all isotypes.

There were several important limitations to our study. Firstly, the symptoms were based on self-report and are potentially affected by subjectivity (misclassification bias), poor recall and desirability bias. Nevertheless, these biases are likely to be consistent across future cohorts unless there are repeated symptom screens in addition to, or in lieu of, retrospective symptom reporting at the time of testing. Secondly, while here we report on sensitivity of antibody testing relative to symptom onset, it is worth noting that the estimated median incubation period for COVID-19 is 5.1 days (95% CI, 4.5 to 5.8 days) [8]. This means that the timing of symptom onset is on average delayed by about five days after the start of infection. Although this is not a limitation of this study per se, it is an important limitation of antibody testing. Thirdly, routine practice at the time, and hence the study, relied on a test (Standard Q COVID-19 IgG/IgM Duo) that performed worse than other antibody tests and was not recommended for marketing authorization in the US. This may have resulted in a particularly poor ability to identify and isolate infectious cases in this context.

Notwithstanding the aforementioned limitations, our study used a community-based cohort of 492 persons with RT-PCR-confirmed COVID-19 to show that the rapid antibody test used in Peru during this time period was a poor substitute for RT-PCR testing; it and other antibody tests detect disease too late to be useful for transmission prevention. At worst, this rapid antibody test could detect less than 50% of infections in the first week of symptoms onset—or approximately the second week since the start of infection — and at best, it could detect the majority of infections by the third week of symptoms onset, which would mean nearly a month after the start of infection.

## Conclusion

Planning for future pandemics requires mechanisms to ensure more rapid and equitable distribution of gold standard diagnostic testing (as well as treatment and prevention/vaccination). The use of an underperforming “rule-in” test—i.e., to guide too little isolation too late—underscores the tragic dilemma facing communities, providers, and health systems in the context of public-health emergencies. The choice is between using imperfect tools—with limited evidence underpinning their performance—to guide timely action or delaying action until and unless strong evidence of high performance emerges. Although WHO and national regulators attempted to provide real-time and high-quality information about performance of diagnostics, treatments, and vaccines, the COVID-19 pandemic exposed major flaws with dire consequences.

## Supplementary Information

**Additional file 1. Table S1. **Reported symptoms of all participants with sample collected (and tested) for serologic antibody test on same day as sample that resulted in RT-PCR positive for SARS-CoV-2 (n=492); no symptom duration outliers excluded. **Table S2. **Reported symptoms of all participants with sample collected (and tested) for serologic antibody test on same day as sample that resulted in RT-PCR positive for SARS-CoV-2 (n=492); mild and extreme symptom duration outliers excluded. **Table S3.** Sensitivity of the rapid antibody test^#^ by symptom duration and outlier exclusion criteria. **Fig. S1.** Plots of antibody test sensitivity against the number of weeks since onset of symptoms among those who reported at least one symptom before testing, using the RT-PCR test as the reference, stratified by the types of outliers excluded in the analysis: extreme outliers, which were defined as persons whose time since symptom onset was longer than the third quartile plus 3 times the interquartile range (IQR, defined as the difference between the first and third quartile of the reported times since onset for a given symptom); mild outliers were those beyond the third quartile plus 1.5 times the IQR); or no outliers excluded. The circles show the crude estimated sensitivity and the error bars show the Wilson’s score 95% confidence intervals. The plotted lines are the fitted segmented linear regressions for the rapid antibody tests. **Table S4.** Segmented regression analyses of antibody test sensitivity by weeks since symptom onset, by symptom duration outlier exclusion criteria. **Table S5. **Overall antibody test sensitivity* per week since symptom onset for common symptoms. **Table S6.**Segmented regression analyses of antibody test sensitivity versus weeks since symptom onset, by symptom.

## Data Availability

The datasets used and/or analysed during the current study are available from the corresponding author on reasonable request.

## References

[1] Sethuraman, Jeremiah SS, Ryo A (2020). Interpreting diagnostic tests for SARS-CoV-2. JAMA.

[2] Deeks JJ, Dinnes J, Takwoingi Y, Davenport C, Spijker R, Taylor-Phillips S (2020). Antibody tests for identification of current and past infection with SARS-CoV-2. Cochrane Database Syst Rev.

[3] Lisboa Bastos M, Tavaziva G, Abidi SK, Campbell JR, Haraoui LP, Johnston JC (2020). Diagnostic accuracy of serological tests for covid-19: systematic review and meta-analysis. BMJ.

[4] Johns Hopkins Coronavirus Resource Center. COVID-19 Map. [Internet]. Baltimore: Johns Hopkins University of Medicine; 2021. https://coronavirus.jhu.edu/map.html. Accessed 23 Aug 2021.

[5] Muggeo VM (2008). Segmented: an R Package to Fit Regression Models with broken-line Relationships. R News.

[6] Santos RE, da Silva MG, Monte Silva MC do, Barbosa DAM, Gomes LdV, Galindo LC et al. Onset and duration of symptoms of loss of smell/taste in patients with COVID-19: A systematic review. Am J Otolaryngol, 2021; 42: 102889. 10.1016/j.amjoto.2020.102889 PMC7833280 33445036

[7] Zhao J, Yuan Q, Wang H, Liu W, Liao X, Su Y (2020). Antibody responses to SARS-CoV-2 in patients with Novel Coronavirus Disease 2019. Clin Infect Dis.

[8] McAloon C, Collins, Hunt K, Barber A, Byrne AW, Butler F (2020). Incubation period of COVID-19: a rapid systematic review and meta-analysis of observational research. BMJ Open.

