# Supplementary material for: Low detection rate of RT-PCR-confirmed COVID-19 using IgM/IgG rapid antibody tests in a large community sample in Lima, Peru
Source: BMC Infect Dis. 2023 Feb 2;23:62. doi: 10.1186/s12879-023-08003-7 (PMC9893955; doi:10.1186/s12879-023-08003-7)
Supplement: Supplementary file 1 — Additional file 1. Table S1. Reported symptoms of all participants with sample collected (and tested) for serologic antibody test on same day as sample that resulted in RT-PCR positive for SARS-CoV-2 (n=492); no symptom duration outliers excluded. Table S2. Reported symptoms of all participants with sample collected (and tested) for serologic antibody test on same day as sample that resulted in RT-PCR positive for SARS-CoV-2 (n=492); mild and extreme symptom duration outliers excluded. Table S3. Sensitivity of the rapid antibody test# by symptom duration and outlier exclusion criteria. Fig. S1. Plots of antibody test sensitivity against the number of weeks since onset of symptoms among those who reported at least one symptom before testing, using the RT-PCR test as the reference, stratified by the types of outliers excluded in the analysis: extreme outliers, which were defined as persons whose time since symptom onset was longer than the third quartile plus 3 times the interquartile range (IQR, defined as the difference between the first and third quartile of the reported times since onset for a given symptom); mild outliers were those beyond the third quartile plus 1.5 times the IQR); or no outliers excluded. The circles show the crude estimated sensitivity and the error bars show the Wilson’s score 95% confidence intervals. The plotted lines are the fitted segmented linear regressions for the rapid antibody tests. Table S4. Segmented regression analyses of antibody test sensitivity by weeks since symptom onset, by symptom duration outlier exclusion criteria. Table S5. Overall antibody test sensitivity* per week since symptom onset for common symptoms. Table S6.Segmented regression analyses of antibody test sensitivity versus weeks since symptom onset, by symptom. [file 12879_2023_8003_MOESM1_ESM.docx]

**Additional file 1**

**Additional file 1:** **Table S1. Reported symptoms of all participants with sample collected (and tested) for serologic antibody test on same day as sample that resulted in RT-PCR positive for SARS-CoV-2 (n=492); no symptoms duration outliers excluded**

|  | **All (n=492)** | **Rapid antibody test result** | | |
| --- | --- | --- | --- | --- |
|  |  | **Negative (n=262)** | **Positive (n=230)** | **p-value*** |
| **Reported having any symptoms (%)** | 380 (77.2) | 209 (79.8) | 171 (74.3) | 0.19 |
| Cough | 252 (51.2) | 133 (50.8) | 119 (51.7) | 0.90 |
| Sore throat | 183 (37.2) | 107 (40.8) | 76 (33.0) | 0.09 |
| Fever | 178 (36.2) | 107 (40.8) | 71 (30.9) | 0.03 |
| Congestion | 131 (26.6) | 79 (30.2) | 52 (22.6) | 0.07 |
| Difficulty breathing | 64 (13.0) | 27 (10.3) | 37 (16.1) | 0.08 |
| Malaise | 181 (36.8) | 103 (39.3) | 78 (33.9) | 25. 2 |
| Diarrhea | 67 (13.6) | 35 (13.4) | 32 (13.9) | 96.2 |
| Vomiting | 53 (10.8) | 24 (9.2) | 29 (12.6) | 27.8 |
| Headache | 167 (33.9) | 93 (35.5) | 74 (32.2) | 49.6 |
| Loss of taste or smell | 28 (5.7) | 12 (4.6) | 16 (7.0) | 34.7 |
| **Median number of reported symptoms, among those reporting (range)** | 3 (1-9) | 3 (1-9) | 3 (1-9) | 0.57 |
| **Median number of days since onset of symptoms, among those reporting (range)** | 7 (1-163) | 6 (1-87) | 10 (2-163) | <0.01 |
| Cough | 7 (1-31) | 5 (1-31) | 9 (2-31) | <0.01 |
| Sore throat | 6 (1-87) | 5 (1-87) | 9 (2-78) | <0.01 |
| Fever | 5 (1-163) | 4 (1-69) | 8 (1-163) | <0.01 |
| Congestion | 6 (1-78) | 5 (1-69) | 9 (1-78) | <0.01 |
| Difficulty breathing | 7 (1-95) | 4 (1-32) | 9 (2-95) | <0.01 |
| Malaise | 6 (1-76) | 5 (1-19) | 8.5 (1-76) | <0.01 |
| Diarrhea | 6 (1-40) | 3 (1-40) | 7.5 (1-28) | <0.01 |
| Vomiting | 4 (1-24) | 3 (1-24) | 7 (2-21) | <0.01 |
| Headache | 5 (1-125) | 5 (1-87) | 7 (1-125) | <0.01 |
| Loss of taste or smell | 5 (2-113) | 3.5 (3-10) | 7.5 (2-113) | 0.02 |
| **Weeks since onset of symptoms, among those reporting (%)** | | | | |
| Week 1 | 220 (57.9) | 155 (74.2) | 65 (38.0) | <0.01 |
| Week 2 | 101 (26.6) | 39 (18.7) | 62 (36.3) |  |
| Week 3 | 31 (8.2) | 7 (3.3) | 24 (14.0) |  |
| Week 4 | 6 (1.6) | 1 (0.5) | 5 (2.9) |  |
| Week 5+ | 22 (5.8) | 7 (3.3) | 15 (8.8) |  |

*Wilcoxon-Mann-Whitney Test for comparing medians, Pearson’s chi-squared test for comparing proportions.

**Additional file 1:** **Table S2. Reported symptoms of all participants with sample collected (and tested) for serologic antibody test on same day as sample that resulted in RT-PCR positive for SARS-CoV-2 (n=492); mild and extreme symptoms duration outliers excluded**

|  | **All (n=492)** | **Rapid antibody test result** | | |
| --- | --- | --- | --- | --- |
|  |  | **Negative (n=262)** | **Positive (n=230)** | **p-value**** |
| **Reported having any symptoms (%)** | 358 (72.8) | 205 (78.2) | 153 (66.5) | <0.01 |
| Cough | 235 (47.8) | 130 (49.6) | 105 (45.7) | 0.43 |
| Sore throat | 170 (34.6) | 105 (40.1) | 65 (28.3) | <0.01 |
| Fever | 163 (33.1) | 104 (39.7) | 59 (25.7) | <0.01 |
| Congestion | 120 (24.4) | 75 (28.6) | 45 (19.6) | 0.03 |
| Difficulty breathing | 57 (11.6) | 26 (9.9) | 31 (13.5) | 27.7 |
| Malaise | 168 (34.1) | 101 (38.5) | 67 (29.1) | 0.04 |
| Diarrhea | 59 (12.0) | 33 (12.6) | 26 (11.3) | 0.76 |
| Vomiting | 48 (9.8) | 23 (8.8) | 25 (10.9) | 0.53 |
| Headache | 159 (32.3) | 89 (34.0) | 70 (30.4) | 0.46 |
| Loss of taste or smell | 27 (5.5) | 12 (4.6) | 15 (6.5) | 0.46 |
| **Median number of reported symptoms, among those reporting (range)** | 3 (1-9) | 3 (1-9) | 3 (1-9) | 0.35 |
| **Median number of days since onset of symptoms, among those reporting (range)** | 5 (2-16) | 3.5 (3-10) | 7 (2-16) | 0.03 |
| Cough | 6 (1-19) | 5 (1-19) | 8 (2-19) | <0.01 |
| Sore throat | 6 (1-19) | 5 (1-18) | 8 (2-19) | <0.01 |
| Fever | 4 (1-15) | 3 (1-10) | 7 (1-15) | <0.01 |
| Congestion | 5 (1-18) | 4 (1-17) | 8 (1-18) | <0.01 |
| Difficulty breathing | 6 (1-19) | 4 (1-15) | 7 (2-19) | <0.01 |
| Malaise | 5.5 (1-16) | 5 (1-16) | 8 (1-16) | <0.01 |
| Diarrhea | 5 (1-16) | 3 (1-9) | 7 (1-16) | <0.01 |
| Vomiting | 4 (1-13) | 3 (1-9) | 5 (2-13) | <0.01 |
| Headache | 5 (1-17) | 5 (1-17) | 7 (1-17) | <0.01 |
| Loss of taste or smell | 5 (2-16) | 3.5 (3-10) | 7 (2-16) | 0.03 |
| **Weeks since onset of symptoms, among those reporting (%)** | | | | |
| Week 1 | 227 (63.4) | 156 (76.1) | 71 (46.4) | <0.01 |
| Week 2 | 109 (30.4) | 42 (20.5) | 67 (43.8) |  |
| Week 3 | 22 (6.1) | 7 (3.4) | 15 (9.8) |  |
| Week 4 | 0 | 0 | 0 |  |
| Week 5+ | 0 | 0 | 0 |  |

*All outliers outside of 1.5 times the IQR of the reported times since onset of symptoms were excluded.

**Wilcoxon-Mann-Whitney Test for comparing medians, Pearson’s chi-squared test for comparing proportions.

| **Additional file 1:** **Table S3.** Sensitivity of the rapid antibody test^#^ by symptom duration outlier exclusion criteria* | | | |
| --- | --- | --- | --- |
| **Group** | **Excluding extreme outliers only (primary analysis)**  **% (95% CI)** | **Excluding mild and extreme outliers**  **% (95% CI)** | **No outliers excluded**  **% (95% CI)** |
| **Presence of reported symptoms** | | | |
| Yes | 44.6 (39.7, 49.7) | 42.7 (37.7, 47.9) | 45.0 (40.1, 50.0) |
| No | 53.3 (44.4, 62.0) | 57.5 (49.0, 65.5) | 52.7 (43.5, 61.7) |
| Yes vs no, p-value** | 0.12 | <0.01 | 0.19 |
| **Weeks since onset of earliest symptom, among those reporting symptoms** | | | |
| Week 1 | 30.4 (24.7, 36.6) | 31.3 (25.6, 37.6) | 29.5 (23.9, 35.9) |
| Week 2 | 62.3 (52.7, 70.9) | 61.5 (52.1, 70.1) | 61.4 (51.6, 70.3) |
| Week 3 | 77.4 (59.8, 88.8) | 68.2 (47.1, 83.7) | 77.4 (59.8, 88.8) |
| Week 4 | 83.3 (41.6, 98.4) | 0 | 83.3 (41.6, 98.4) |
| Week 5+ | 60.0 (23.1, 88.0) | 0 | 68.2 (47.1, 83.7) |
| Comparing time intervals, p-value*** | <0.01 | <0.01 | <0.01 |

^#^For purposes of calculating sensitivity, antibody tests that are positive for IgG, IgM, or both are considered positive. *The primary analysis excluded extreme outliers; i.e., persons whose time since symptom onset was longer than the third quartile plus 3 times the interquartile range (IQR, defined as the difference between the first and third quartile of the reported times since onset for a given symptom). In sensitivity analyses, we explored whether results were sensitive to the exclusion of mild outliers (i.e., beyond the third quartile plus 1.5 times the IQR) or inclusion of all outliers.


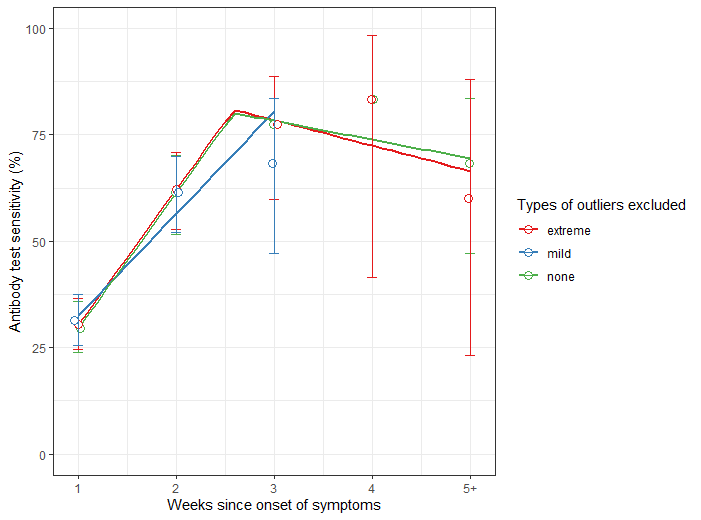


**Additional file 1:** **Fig. S1.** Plots of antibody tests’ sensitivity against the number of weeks since onset of symptoms among those who reported at least one symptom before testing, using the RT-PCR test as the reference, stratified by the types of outliers excluded in the analysis: extreme outliers, which were defined as persons whose time since symptom onset was longer than the third quartile plus 3 times the interquartile range (IQR, defined as the difference between the first and third quartile of the reported times since onset for a given symptom); mild outliers were those beyond the third quartile plus 1.5 times the IQR); or no outliers excluded. The circles show the crude estimated sensitivity and the error bars show the Wilson’s score 95%CI confidence intervals. The plotted lines are the fitted segmented linear regressions for the rapid antibody tests.

**Additional file 1:** **Table S4. Segmented regression analyses of antibody test sensitivity by weeks since symptom onset, by symptom duration outlier exclusion criteria**

| **Outliers excluded*** | **% change per week before breakpoint (95%CI)** | **Breakpoint (95%CI)** | **% change per week after breakpoint (95%CI)** | **Segmented regression model R-squared (%)** |
| --- | --- | --- | --- | --- |
| Extreme only (primary analysis) (n=372) | 31.9 (24.8, 39.0) | 2.6 (2.1, 3.0) | -6.0 (-25.7, 13.7) | 96.9 |
| Mild and extreme (n=358)** | 24.0 (10.4, 37.7) | n/a | n/a | 84.5 |
| None (n=380) | 31.8 (26.1, 37.6) | 2.6 (2.2, 2.9) | -4.4 (-17.7, 8.9) | 98.2 |

*Extreme outliers were defined as persons whose time since symptom onset was longer than the third quartile plus 3 times the interquartile range (IQR, defined as the difference between the first and third quartile of the reported times since onset for a given symptom). Mild outliers were those beyond the third quartile plus 1.5 times the IQR).

**No breakpoint estimated

**Additional file 1:** **Table S5. Overall antibody test sensitivity* per week since symptom onset for common symptoms**

|  | **Weeks since symptom onset** | | | | | | | | | |
| --- | --- | --- | --- | --- | --- | --- | --- | --- | --- | --- |
| **Symptom** | 1 | | 2 | | 3 | | 4 | | 5+ | |
|  | n | Test sensitivity (%, 95%CI) | n | Test sensitivity (%, 95%CI) | n | Test sensitivity (%, 95%CI) | n | Test sensitivity (%, 95%CI) | n | Test sensitivity (%, 95%CI) |
| Cough | 181 | 34.8 (28.3, 42.0) | 97 | 60.8 (50.9, 69.9) | 44 | 54.5 (68.3, 40.1) | 30 | 46.7 (30.3. 63.8) | 0 | n/a |
| Sore throat | 129 | 26.4 (19.5, 34.6) | 63 | 47.6 (35.8, 59.7) | 31 | 48.4 (32.0, 65.1) | 21 | 38.1 (20.8, 59.2) | 0 | n/a |
| Fever | 136 | 27.9 (21.1, 36.1) | 37 | 78.4 (62.5, 88.8) | 15 | 80.0 (53.9, 93.5) | 7 | 85.7 (46.4, 99.0) | 0 | n/a |
| Congestion | 98 | 26.5 (18.8, 36.1) | 38 | 50.0 (34.9, 65.1) | 24 | 54.2 (35.1, 72.0) | 14 | 35.7 (16.4, 61.4) | 18 | 38.9 (20.4, 61.5) |
| Difficulty breathing | 47 | 40.4 (27.7, 54.7) | 26 | 57.7 (38.9, 74.4) | 16 | 50.0 (28.1, 71.9) | 11 | 36.4 (15.2, 64.8) | 11 | 36.4 (15.2, 64.8) |
| Malaise | 128 | 28.1 (21.1, 36.5) | 57 | 57.9 (45.0, 70.0) | 25 | 56.0 (37.1, 73.3) | 13 | 38.5 (17.8, 64.6) | 0 | n/a |
| Diarrhea | 50 | 36.0 (24.2, 49.9) | 16 | 68.8 (44.1, 85.9) | 9 | 77.8 (44.1, 94.3) | 3 | 50.0 (19.0, 81.0) | 0 | n/a |
| Vomiting | 43 | 39.5 (26.4, 54.5) | 17 | 47.1 (26.3, 69.0) | 10 | 40.0 (16.9, 68.8) | 0 | n/a | 0 | n/a |
| Headache | 129 | 34.1 (26.5, 42.7) | 51 | 51.0 (37.7, 64.1) | 24 | 33.3 (18.0, 53.5) | 0 | n/a | 0 | n/a |
| Loss of taste or smell | 18 | 44.4 (24.6, 66.3) | 8 | 75.0 (40.0, 93.3) | 1 | 100 (17.1, 100) | 0 | n/a | 0 | n/a |

^*^For purposes of calculating sensitivity, antibody tests that are positive for IgG, IgM, or both are considered positive.

**Additional file 1:** **Table S6. Segmented regression analyses of antibody test sensitivity versus weeks since symptom onset, by symptom**

| **Symptom** | **% change per week before breakpoint (95%CI)** | **Breakpoint (week, 95%CI)** | **% change per week after breakpoint (95%CI)** | **Segmented regression model adjusted R-squared (%)** |
| --- | --- | --- | --- | --- |
| Cough | 31.7 (27.6, 35.8) | 2.1 (1.3,2.9) | -10.4 (-35.2, 14.3) | 98.7 |
| Sore throat | 24.7 (22.3, 27.0) | 2.3 (1.8, 2.8) | -10.3 (-24.2, 0.0) | 99.3 |
| Fever | 33.8 (22.8, 44.8) | 2.2 (0, 5.9) | 5.7 (-82.0, 93.5) | 92.5 |
| Congestion | 25.6 (22.4, 28.8) | 2.3 (1.8, 2.7) | -8.0 (-18.3, 2.2) | 98.8 |
| Difficulty breathing | 39.9 (35.8, 44.0) | 1.5 (1.4, 1.7) | -8.3 (-17.1, 0.0) | 99.6 |
| Malaise | 28.7 (27.9, 29.4) | 2.4 (2.2, 2.5) | -17.5 (-22.8, -12.3) | 99.9 |
| Diarrhea | 35.1 (33.5, 36.7) | 2.6 (2.4, 2.7) | -27.8 (-37.2, -18.3) | 99.9 |
| Vomiting* | 38.7 (25.5, 51.9) | n/a | n/a | -66.3 |
| Headache* | 32.0 (0, 63.2) | n/a | n/a | -69.9 |
| Loss of taste/smell* | 15.2 (10.7, 19.7) | n/a | n/a | 99.5 |

*no breakpoint was estimated
